# Anesthetic Management of a Patient With Prinzmetal Angina

**DOI:** 10.7759/cureus.41857

**Published:** 2023-07-13

**Authors:** Cristina P Sousa, Filipa Sales, Francisco Teixeira, Daniel Seabra, Mariana Cunha

**Affiliations:** 1 Anesthesiology Department, Centro Hospitalar Trás-os-Montes e Alto Douro, Vila Real, PRT; 2 Anesthesiology Department, Hospital Pedro Hispano, Matosinhos, PRT; 3 Cardiology Service, Medicine Department, Hospital Pedro Hispano, Matosinhos, PRT

**Keywords:** variant angina, anesthetic management, myocardial ischemia, coronary artery spasm, prinzmetal angina

## Abstract

Prinzmetal angina (PA) is characterized by the development of reversible vasoconstriction of the coronary arteries, transient ischemic electrocardiographic changes in the ST segment, chest pain at rest, and prompt response to nitrates.

Spasms of the coronary arteries can be precipitated during the perioperative period by an imbalance of vasodilator and vasoconstrictor factors of smooth muscle cells, which can lead to myocardial ischemia, cardiac arrhythmias, and death. Nevertheless, this is a relatively unrecognized topic, and literature is scarce about it.

We present a case report detailing the successful anesthetic management of a patient diagnosed with PA and a documented nitrate allergy, who underwent bilateral ureterorenoscopy.

## Introduction

Prinzmetal angina (PA) or variant angina, first described by Prinzmetal et al. in 1959, is characterized by transient and reversible vasoconstriction of the coronary arteries and consequent myocardial ischemia [1]. The coronary arteries may develop spasms as a result of increased reactivity of the vessels to several precipitating factors. The cause of coronary hyperreactivity is unclear but could be related to endothelial dysfunction and impaired regulatory mechanism for vasoconstriction and vasodilation [2]. The balance between sympathetic and parasympathetic tone is an important factor in regulating coronary circulation. Imbalances can lead to exaggerated vasoconstriction even in normal circumstances, restricting blood flow and potentially causing myocardial ischemia. This condition is an underestimated cause of acute coronary syndromes, malignant arrhythmias, and sudden cardiac death [2,3].

Case reports describing perioperative coronary artery spasms have been published in patients with or without a history of coronary artery disease undergoing general or regional anesthesia [4-7]. However, the overall incidence of coronary artery spasm events occurring in the perioperative period is unknown. Perioperative coronary artery spasm seems to occur more frequently under combined general and epidural anesthesia [6]. The reflex sympathetic activity above the level of the sympathetic blockade could be the responsible mechanism for coronary artery spasm [7].

Our case report presents a successful anesthetic approach for a patient with a history of PA and a documented allergy to nitrates, who underwent bilateral ureterorenoscopy.

## Case presentation

**Figure 1:**
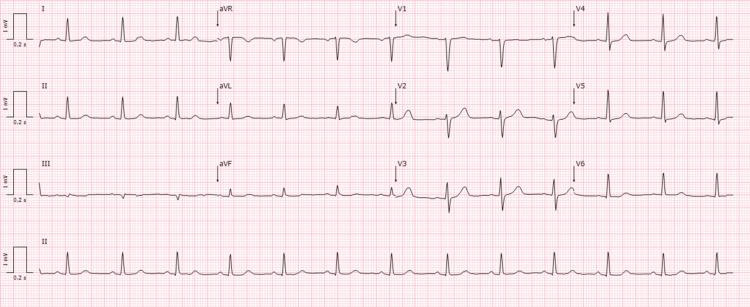
Preoperative electrocardiogram. Normal sinus rhythm of 70 beats/minute with normal axis and intervals.

A 67-year-old, 82 kg, 170 cm (body mass index: 28.4 kg/m^2^), American Society of Anesthesiologists (ASA) III male was proposed for bilateral ureterorenoscopy due to ureteral lithiasis. The patient's past medical history included cardiovascular risk factors, such as arterial hypertension, dyslipidemia, and overweight, and documented PA, with normal left ventricle function. The patient was chronically medicated with telmisartan and hydrochlorothiazide, nifedipine, atorvastatin, and acetylsalicylic acid. Importantly, the patient had a documented anaphylactic reaction following nitrate administration. His physical examination revealed no abnormalities, and a preoperative electrocardiogram (ECG) demonstrated sinus rhythm without abnormalities (Figure 1).

The diagnosis of PA was based on clinical criteria and transient ischemic changes on the ECG. The patient was asymptomatic from a cardiovascular point of view until October 2007, when he began a clinical condition characterized by multiple episodes of chest pain radiating to the left upper limb, at rest, which reversed spontaneously and lasted less than one minute. Subsequently, a 24-hour Holter was performed, which revealed transient ST segment elevation during the spontaneous episodes. A coronary angiography was also performed, and the presence of significant coronary disease was ruled out.

Considering the patient's medical history, a multidisciplinary approach was adopted, involving consultation with the cardiology team. It was determined that ensuring the availability of intravenous calcium channel blockers (verapamil) was crucial in managing potential vasospasm episodes during the perioperative period.

During the intraoperative period, processed electroencephalogram, neuromuscular block monitoring, and invasive blood pressure were used in addition to standard monitoring. The anesthetic approach consisted of a balanced general anesthesia. Infusion of remifentanil was guided by a target-controlled infusion system (TCI) (Minto model) with an effect-site target concentration of 2 ng ml-1. Orotracheal intubation was performed with a 7.5 tube after the administration of 120 mg of propofol and 50 mg of rocuronium. Anesthesia was maintained with sevoflurane and remifentanil. Normothermia and normocapnia were carefully monitored throughout the entire procedure. The analgesia regimen included 1 g acetaminophen and 100 mg tramadol. Ondansetron 4 mg was administered for nausea and vomiting prophylaxis.

At the end of the procedure, neuromuscular blockade was reversed with sugammadex. When the train-of-four (TOF) ratio exceeded 90%, the patient was extubated, and remifentanil infusion was stopped. The procedure lasted one hour, with no hemodynamic or electrocardiographic events to record, especially ST segment changes that could suggest acute myocardial ischemia. The patient was transferred to the postanesthesia care unit, where he remained comfortable and asymptomatic. Subsequently, the patient was admitted to the level 2 intensive care unit for monitoring during the first 24 hours postoperatively. The hospital stay was without any complications, and the patient was discharged home on the third day after the surgery.

## Discussion

PA is an underestimated cause of myocardial infarction. It is characterized by transient vasoconstriction of the coronary arteries caused by an imbalance between the vasodilator and vasoconstrictor response of the coronary circulation [3]. The clinical presentation of the condition is diverse but commonly involves chest pain occurring at rest, particularly during nighttime or early morning. These episodes typically exhibit a prompt response to the administration of nitrates. PA disease appears to have a higher prevalence among Caucasian males aged between 40 and 70 years, particularly among smokers [2]. Several precipitating factors have been identified, including physical and/or mental stress, alcohol consumption, cold exposure, hyperventilation, Valsalva maneuver, cocaine use, sympathomimetics, beta-blockers, cholinergic agents, and general anesthesia [4,5,8].

The mechanisms underlying the development of PA are still poorly defined and are probably multifactorial. Endothelial dysfunction may play a role in the pathophysiology. A healthy endothelium responds to acetylcholine through vasodilation via the release of nitric oxide, while a dysfunctional endothelium does not respond to acetylcholine, thus facilitating the action of vasoconstrictor mediators [3]. On the other hand, acetylcholine is, by itself, a vasoactive substance capable of causing vasospasm via muscarinic receptors in vascular smooth muscle cells. This mechanism may explain why several endothelium-dependent vasodilators, such as acetylcholine, ergonovine, histamine, and serotonin, cause vasoconstriction in patients with PA, highlighting the role of endothelium-independent vasodilators (nitrates) in the treatment of vasospasm [9].

During the perioperative period, and specifically in patients undergoing general anesthesia, there are several precipitating factors capable of causing coronary artery spasms. Kishimoto et al. [10] reported a case in which ST segment elevation occurred after the administration of α-adrenergic agonist vasopressors (ephedrine and phenylephrine) to manage hypotension in an 80-year-old female scheduled to undergo a complex maxillofacial procedure. The patient had no history of coronary artery disease. Miyoshi et al. [11] described a case of a 66-year-old male with no history of ischemic heart disease, proposed for cervical lymph node dissection, who developed coronary artery spasm induced by cervical manipulation and consequent increased vagal tone. Other reports have described hypotension, the administration of anticholinergic drugs, hyperventilation, allergic reaction, and inadequate depth of anesthesia as possible causes of coronary artery spasm [6]. Regarding the choice of anesthetic agent for noncardiac surgery, there seems to be no difference in terms of patient outcome, since the incidence of perioperative cardiac events seems not to be influenced by the choice of a volatile or an intravenous anesthetic regimen [12].

In our clinical report, the goal was to reduce exogenous and endogenous catecholamine exposure, ensuring a smooth induction using remifentanil and low-dose propofol titration and guaranteeing adequate depth of anesthesia for the surgical stimulus. The anesthetic depth was monitored and ensured throughout the procedure. Remifentanil was chosen because of its efficacy for obtunding response to noxious stimuli such as orotracheal intubation and surgical stress. In this case, ureteral dilation by fluid instillation may increase vagal tone. Continuous infusion of remifentanil during the extubation period was also used to suppress stress and coughing responses and improve patients' comfort during emergence. Significant systemic stress responses to pain and airway stimulation can occur during this period, which could cause a vasospastic episode. In line with the above, Lindsay et al. [13] reported a 60-year-old transgender woman presented for gender reassignment surgery, with a previous diagnosis of coronary vasospasm, who developed ST elevation and wide complex tachycardia in a probable context of coronary vasospasm upon extubation. The authors considered that extubation triggered the release of catecholamines with subsequent vasospasm, leading to ST segment elevation and ventricular tachycardia.

The diagnosis of PA is based on three considerations: clinical presentation, evidence of transient ischemia on the ECG during the angina episode, and demonstration of spontaneous or provoked coronary vasospasm by coronary angiography [14]. Since patients are unconscious and cannot complain of symptoms during general anesthesia, ST segment elevation on ECG could be the only clue for recognizing coronary vasospasm, evidence of the need for high clinical suspicion for prompt management. Perioperative echocardiography could also be a useful tool for the detection of myocardial ischemia in these patients, allowing for timely intervention. During surgery, the anesthesiologist could use echocardiography to evaluate regional wall motion abnormalities, which can be indicative of ischemia in specific areas of the heart. Reduced or absent wall motion in certain segments of the myocardium may suggest compromised blood flow to those regions. In addition to wall motion analysis, perioperative echocardiography can also assess other parameters related to myocardial ischemia, namely, left ventricular ejection fraction, diastolic function, and the presence of regional wall thickening abnormalities.

Treatment of PA consists of lifestyle modification, avoiding factors that perpetuate endothelial dysfunction, and pharmacological therapy with calcium channel blockers and nitrates [15]. Calcium channel blockers inhibit the influx of calcium into smooth muscle cells and stimulate the production of nitric oxide, leading to vasodilation. Nitrates lead to vasodilation upon conversion to nitric oxide, making them very effective in the immediate relief of angina. In contrast to traditional exertional angina, beta-blockers are believed to worsen symptoms through an increased vasoconstrictor response [16]. In the case presented here, the patient had a documented allergy to nitrates, so the available option to reverse possible vasospasm would be intravenous administration of calcium channel blockers. The possibility of prophylactic administration of a calcium channel blocker was discussed with cardiology. However, due to the risk of myocardial depression and hypotension, it was decided to ensure the availability of the drug intraoperatively and its use only if necessary.

**Figure 2:**
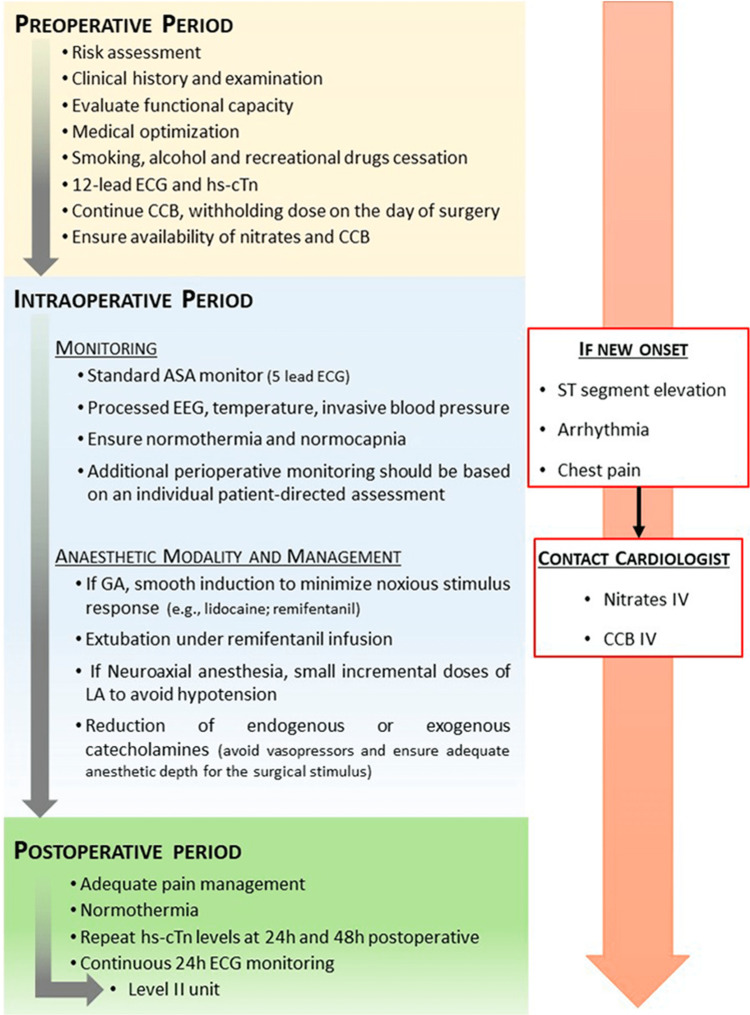
Approach to coronary artery spasm during the perioperative period. ASA: American Society of Anesthesiologists; CAS: coronary artery spasm; CCB: calcium channel blockers; ECG: electrocardiogram; EEG: electroencephalogram; GA: general anesthesia; hs-cTn: high-sensitive cardiac troponin; LA: local anesthetic

Due to the lack of evidence on how to approach these patients, the authors suggest an algorithm (Figure 2).

## Conclusions

In conclusion, this case report demonstrates the successful anesthetic management of a patient with PA and a documented nitrate allergy undergoing bilateral ureterorenoscopy. The implementation of a multidisciplinary approach, involving cardiology consultation, along with the availability of intravenous calcium channel blockers, and meticulous attention to smooth induction and extubation were pivotal in achieving a positive perioperative outcome. These findings further emphasize the significance of customized anesthetic strategies and alternative medication options for patients with Prinzmetal angina and nitrate allergies, thus contributing valuable insights to the existing literature.

## References

[1] Prinzmetal M, Kennamer R, Merliss R, Wada T, Bor N (1959). Angina pectoris. I. A variant form of angina pectoris; preliminary report. Am J Med.

[2] Yasue H, Nakagawa H, Itoh T, Harada E, Mizuno Y (2008). Coronary artery spasm--clinical features, diagnosis, pathogenesis, and treatment. J Cardiol.

[3] Lanza GA, Careri G, Crea F (2011). Mechanisms of coronary artery spasm. Circulation.

[4] Peng W, Huang S, Zhou S, Yang N, Zuo M (2018). Case report: life-threatening coronary artery spasm under transversus abdominis plane block in combination with general anesthesia. BMC Anesthesiol.

[5] Sidi A, Dahleen L, Gaspardone A (2008). Coronary vasospasm during anesthesia induction: awareness, recognition, possible mechanisms, anesthetic factors, and treatment. J Clin Anesth.

[6] Koshiba K, Hoka S (2001). Clinical characteristics of perioperative coronary spasm: reviews of 115 case reports in Japan. J Anesth.

[7] Easley RB, Rosen RE, Lindeman KS (2003). Coronary artery spasm during initiation of epidural anesthesia. Anesthesiology.

[8] Takaoka K, Yoshimura M, Ogawa H (2000). Comparison of the risk factors for coronary artery spasm with those for organic stenosis in a Japanese population: role of cigarette smoking. Int J Cardiol.

[9] Sandoo A, van Zanten JJ, Metsios GS, Carroll D, Kitas GD (2010). The endothelium and its role in regulating vascular tone. Open Cardiovasc Med J.

[10] Kishimoto N, Kato M, Nakanishi Y, Hasegawa A, Momota Y (2018). Recurrent coronary artery spasm induced by vasopressors during two operations in the same patient under general anesthesia. Anesth Prog.

[11] Miyoshi H, Saeki N, Nakamura R, Kurita S, Kawamoto M (2012). A case of coronary artery spasm caused by manipulation of the neck: heart rate variability analysis. J Anesth.

[12] Lurati Buse GA, Schumacher P, Seeberger E (2012). Randomized comparison of sevoflurane versus propofol to reduce perioperative myocardial ischemia in patients undergoing noncardiac surgery. Circulation.

[13] Lindsay PJ, Frank RC, Bittner EA, Berg S, Chang MG (2020). St elevations and ventricular tachycardia secondary to coronary vasospasm upon extubation. Case Rep Anesthesiol.

[14] Beltrame JF, Crea F, Kaski JC (2017). International standardization of diagnostic criteria for vasospastic angina. Eur Heart J.

[15] Harris JR, Hale GM, Dasari TW, Schwier NC (2016). Pharmacotherapy of vasospastic angina. J Cardiovasc Pharmacol Ther.

[16] Takahashi J, Nihei T, Takagi Y (2015). Prognostic impact of chronic nitrate therapy in patients with vasospastic angina: multicentre registry study of the Japanese coronary spasm association. Eur Heart J.

